# Cellular and Molecular Mechanisms of Non-Invasive Brain Stimulation Techniques: A Systematic Review on the Implications for the Treatment of Neurological Disorders

**DOI:** 10.3390/cells14241996

**Published:** 2025-12-15

**Authors:** Valerio Sveva, Marco Mancuso, Alessandro Cruciani, Elias Paolo Casula, Giorgio Leodori, Silvia Antonella Selvaggi, Matteo Bologna, Vincenzo Di Lazzaro, Anna Latorre, Lorenzo Rocchi

**Affiliations:** 1 Department of Human Neuroscience, University of Rome “Sapienza”, Viale dell’Università 30, 00185 Rome, Italy; valerio.sveva@uniroma1.it (V.S.); marco.mancuso@uniroma1.it (M.M.); giorgio.leodori@uniroma1.it (G.L.); matteo.bologna@uniroma1.it (M.B.); 2 Unit of Neurology, Neurophysiology, Neurobiology, and Psychiatry, Department of Medicine and Surgery, Università Campus Bio-Medico di Roma, Via Alvaro del Portillo 21, 00128 Rome, Italy; alessandro.cruciani@unicampus.it (A.C.); sa.selvaggi@unicampus.it (S.A.S.); v.dilazzaro@unicampus.it (V.D.L.); 3 Department of System Medicine, University of Tor Vergata, Via Cracovia 50, 00133 Rome, Italy; elias.casula@gmail.com; 4 IRCCS Neuromed, Via Atinense 18, 86077 Pozzilli, Italy; 5 Department of Clinical and Movement Neurosciences, UCL Queen Square Institute of Neurology, University College London, London WC1E 6BT, UK; 6 Department of Medical Sciences and Public Health, University of Cagliari, Cittadella Universitaria di Monserrato, Blocco I S.S. 554 bivio per Sestu, Monserrato, 09042 Cagliari, Italy; l.rocchi@ucl.ac.uk

**Keywords:** rTMS, TBS, PAS, tDCS, tACS, LTP-like, LTD-like plasticity, gene expression, molecular mechanism, cellular mechanism

## Abstract

**Highlights:**

**What are the main findings?**

- NIBS effects involve multiple, interacting plasticity pathways that extend beyond a simple LTP/LTD framework
- Across studies, NIBS modulates BDNF–TrkB signaling, neurotransmitter receptor activity (NMDA/AMPA, GABA), and calcium-dependent mechanisms supporting synaptic plasticity

**What are the implications of the main findings?**

- Mechanistic biomarkers—rather than MEP-based LTP/LTD interpretations alone—are needed to guide and optimize NIBS protocols
- The involvement of conserved plasticity pathways supports the translational potential of NIBS for CNS disorders, although further cross-species validation is required

**Abstract:**

Non-invasive brain stimulation (NIBS) techniques—including repetitive transcranial magnetic stimulation (rTMS), theta-burst stimulation (TBS), paired associative stimulation (PAS), transcranial direct current stimulation (tDCS), and transcranial alternating current stimulation (tACS)—have emerged as valuable tools for modulating neural activity and promoting plasticity. Traditionally, their effects have been interpreted within a binary framework of long-term potentiation (LTP)-like and long-term depression (LTD)-like plasticity, largely inferred from changes in motor evoked potentials (MEPs). However, existing models do not fully capture the complexity of the biological processes engaged by these techniques and despite extensive clinical application, the cellular and molecular mechanisms underlying NIBS remain only partially understood. This systematic review, conducted in accordance with the PRISMA 2020 guidelines, synthesizes evidence from in vivo, in vitro, and ex vivo studies to delineate how NIBS influences neurotransmission through intracellular signaling, gene expression, and protein synthesis at the cellular level. Emphasis is placed on the roles of classical synaptic models, grounded in Ca^2+^-dependent glutamatergic signaling and receptor phosphorylation dynamics, as well as broader forms of plasticity involving BDNF–TrkB signaling, epigenetic modifications, neuroimmune and glial interactions, anti-inflammatory pathways, and apoptosis- and survival-related cascades. By integrating findings in humans with those in animal and cellular models, we identify both shared and technique-specific molecular mechanisms underlying NIBS-induced effects, highlighting emerging evidence for multi-pathway, non-binary plasticity mechanisms. Understanding these convergent pathways provides a mechanistic foundation for refining stimulation paradigms and improving their translational relevance for treatment of neurological and psychiatric disorders.

## 1. Introduction

Non-invasive brain stimulation (NIBS) techniques have become established tools for modulating brain activity by applying electrical or electromagnetic stimuli to the scalp [1]. Initially developed for research purposes, they are now widely used in both experimental and clinical contexts to probe and influence neural circuits, thereby providing key insights into brain function and plasticity [2]. The term non-invasive brain stimulation (NIBS) encompasses several techniques, such as repetitive transcranial magnetic stimulation (rTMS)—including variants like theta burst stimulation (TBS) and paired associative stimulation (PAS)—as well as transcranial electrical stimulation (tES), most commonly implemented as transcranial direct current stimulation (tDCS) or transcranial alternating current stimulation (tACS) [3]. Over the past decades, interest in these methods has steadily grown, fueled by their relative ease of use, favorable safety profile, and promising translational applications in the treatment of central nervous system (CNS) disorders [4]. Clinical studies have explored their therapeutic potential across a wide spectrum of conditions, ranging from depression [5], Parkinson’s disease [6] and Alzheimer’s disease (AD) [7,8], to stroke [9], chronic pain [10], epilepsy [11], and migraine [12].

Large, randomized, placebo-controlled studies have shown that NIBS techniques can induce not only immediate but also long-lasting modifications in brain function, with effects persisting well beyond the stimulation period [4]. These sustained outcomes are thought to arise from mechanisms of synaptic plasticity, i.e., the brain’s ability to remodel neural connections in response to external stimuli [13]. Synaptic plasticity can be broadly classified into short-term synaptic plasticity (STSP), which supports rapid information processing, and long-term synaptic plasticity (LTSP), which underlies learning, memory, and adaptive behaviors [14]. STSP primarily depends on presynaptic mechanisms such as alterations in neurotransmitter release, whereas LTSP involves enduring structural and molecular modifications, including dendritic spine remodeling [15]. LTSP is commonly conceptualized within a binary framework of long-term potentiation (LTP)—associated with enhanced synaptic efficacy—and long-term depression (LTD)—associated with reduced efficacy [16,17]. Among the key principles governing these processes is Hebbian plasticity, based on the notion “cells that fire together, wire together,” which drives activity-dependent strengthening of synapses [18]. To maintain network stability, homeostatic plasticity acts as a counterbalance, globally adjusting synaptic strengths to preserve overall excitatory–inhibitory balance [19]. Metaplasticity, in turn, modulates the synaptic change by altering the thresholds for potentiation or depression, thereby shaping how neurons adapt to future activity [19,20].

In human studies, NIBS-induced changes are typically evaluated through behavioral measures [21,22,23,24], such as reaction times [25], or through physiological recordings with electroencephalography (EEG) [26] and electromyography (EMG). Among these, the motor evoked potential (MEP) elicited by single-pulse TMS has emerged as a central biomarker, widely used to quantify NIBS-related plasticity within the LTP/LTD framework [27]. However, MEPs provide an indirect and at times ambiguous readout, as their interpretation is confounded by factors such as spinal excitability and pronounced inter-individual variability [28]. Hence, there is a risk of interpreting NIBS effects within an overly dichotomous framework of LTP-like and LTD-like plasticity, which may obscure the complexity of the underlying neural processes [26].

Despite the widespread use of the LTP-like and LTD-like framework, the underlying NIBS mechanisms remain only partially understood, especially in humans where these cannot be directly verified. Significant evidence suggests that plasticity arises from a dynamic interplay of processes that include calcium (Ca^2+^) signaling, neurotransmitter release, gene expression, protein synthesis, and neuro-glial interactions [29,30]. Central to these processes are NMDA receptors (NMDARs), which mediate Ca^2+^ influx during membrane depolarization and trigger intracellular cascades required for the induction of LTP [31,32]. AMPA receptors (AMPARs) are equally critical, particularly through their trafficking and clustering at postsynaptic sites, which shape the expression of synaptic plasticity [17]. Beyond glutamatergic transmission, modulatory influences such as neurotrophins—including brain-derived neurotrophic factor (BDNF) [33,34]—and epigenetic mechanisms such as histone acetylation [35] further regulate synaptic strength and neuronal adaptability. Glial cells also play an active role: astrocytes and microglia contribute to synaptogenesis [36,37], synaptic pruning [38,39], and neurotransmitter homeostasis [40,41]. Nevertheless, key questions remain. For example, it is still unclear whether magnetic or electrical stimulation directly modulates excitatory or inhibitory synaptic strength at the single-cell level, and the translational relevance of findings from animal models to human physiology remains limited. These gaps highlight the need for an integrated framework linking cellular and molecular mechanisms to the macroscopic effects observed in humans.

In this review, we present an updated perspective on the cellular and molecular foundation bases of magnetic and electrical NIBS, outlining how these techniques engage shared or distinct pathways of synaptic plasticity, evaluating the current evidence supporting these mechanisms, and considering the implications for future experimental and translational research. While NIBS is increasingly explored as a potential therapeutic tool in neurological disorders, this review focuses specifically on the underlying biomolecular mechanisms of plasticity and does not infer clinical efficacy, which remains to be established. By clarifying these mechanistic underpinnings, our aim is to support the development of more precise research hypotheses and contribute to the responsible advancement of NIBS as a therapeutic strategy for CNS disorders.

## 2. Materials and Methods

The study is conducted following the PRISMA Statement, and the Review was not registered in any open registry database. A comprehensive search was performed across PubMed/MEDLINE, Scopus, Google Scholar, and Google, independently by three reviewers (V.S., M.M., A.C.), covering all records from inception to January 2025.

Search terms related to rTMS, TBS, PAS, tDCS, and tACS were combined using Boolean operators (OR/AND) as shown in Table 1, and reference lists of eligible papers were screened for additional studies.

Eligible studies included in vivo ex vivo, or in vitro investigations of cellular or molecular mechanisms of NIBS in healthy models or models of neurological or psychiatric disease. Reviews, pilot, and retrospective studies were excluded, as were those lacking clear stimulation parameters or relying solely on indirect physiological data.

After removing duplicates, records that were irrelevant to the topic, of low methodological quality, or lacking cellular or molecular outcomes were excluded based on title and abstract screening. Titles and abstracts were screened independently, and full texts assessed for eligibility. Only English-language original studies were included.

**Figure 1:**
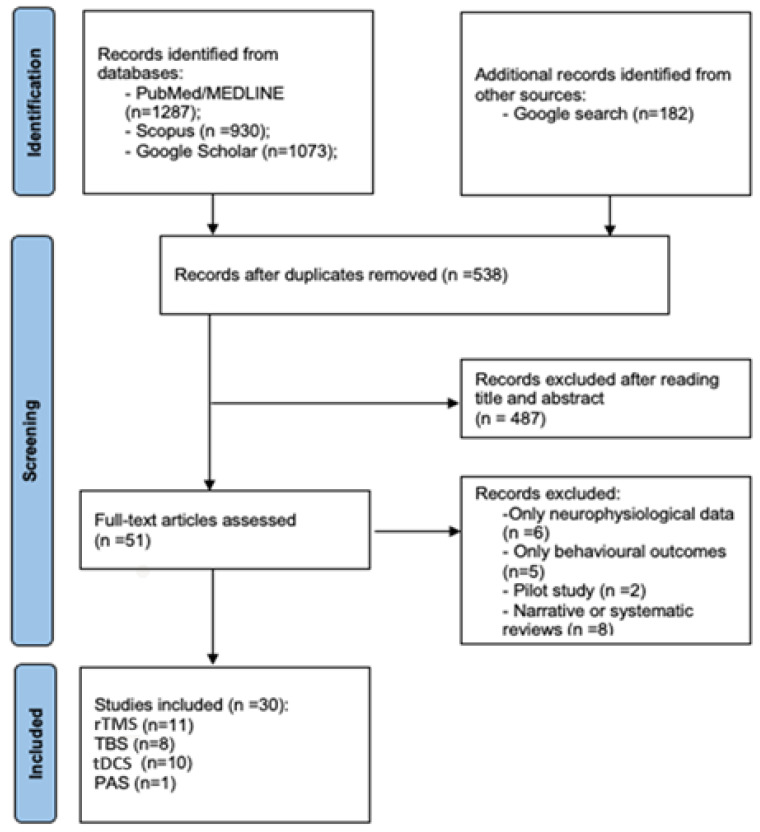
PRISMA flow diagram of retrieved studies.

Disagreements were resolved by discussion among the reviewers and senior authors. The study-selection process is summarized in Figure 1 (PRISMA flow diagram). For each included study, data on model, stimulation parameters, molecular targets, and main results were extracted and summarized narratively in Table 2, Table 3 and Table 4. A formal quantitative risk-of-bias analysis was not performed, but methodological strengths and limitations were qualitatively noted.

## 3. Results

A comprehensive literature search was conducted across PubMed/MEDLINE, Scopus, and Google Scholar, yielding a total of 3290 records (PubMed/MEDLINE: 1287; Scopus: 930; Google Scholar: 1073). An additional 182 records were identified through a supplementary Google search. After removing duplicates, 538 records remained for title and abstract screening. Of these, 487 records were excluded due to lack of relevance to the research question. Fifty-one full-text articles were then assessed for eligibility. After a full-text review, 21 articles were excluded for the following reasons: 6 reported only neurophysiological outcomes, 5 reported only behavioral outcomes, 2 were pilot studies, and 8 were narrative or systematic reviews.

A total of 30 studies met the inclusion criteria and were included in the qualitative synthesis, as summarized in Figure 1 (PRISMA flow diagram).

In this section, the content is organized according to the stimulation techniques employed, each introduced by a brief overview of the methodological principles underlying studies conducted in humans. We then analyze cellular and molecular investigations, highlighting, where possible, translational work in which findings from model systems have been extended to human applications. Table 2 summarizes rTMS studies, Table 3 presents those using TBS and PAS, and Table 4 lists investigations based on tDCS.

Across the studies included, considerable heterogeneity was present in stimulation protocols, model systems, and methodological designs. Stimulation parameters varied widely, ranging from low- (0.5–1 Hz) to high-frequency (10–20 Hz) rTMS/rMS, with large differences in total dose, session duration, pulse number, and intensity (15–120% of motor threshold or MSO). Experimental models also differed substantially, spanning in vitro neuronal and cell-line preparations, ex vivo hippocampal slices, and diverse in vivo rodent models, including ischemia–reperfusion injury, CUMS-induced depression, stroke (MCAO), and Alzheimer’s disease. Sample sizes and grouping strategies ranged from small in vitro studies with a few conditions to large multi-group animal experiments exceeding 100 subjects. Methodological variability further contributed to the overall heterogeneity of the evidence base. The most frequently applied cellular and molecular techniques included immunohistochemistry (IHC, n = 13), Western blotting (n = 12), and quantitative real-time polymerase chain reaction (qRT-PCR, n = 11). Additional approaches comprised ELISA (n = 6), immunofluorescence (IF, n = 5), immunocytochemistry (ICC, n = 4), transcriptome analysis (n = 4), microarray analysis (n = 2), in situ hybridization (n = 1), metabolomic profiling (n = 1), and microdialysis (n = 1).

### 3.1. TMS Protocols

In human studies, TMS delivers a transient magnetic field to the scalp through a coil, inducing depolarization of cortical neurons [72]. When applied repetitively using specific stimulation protocols, it can modulate neural activity, leading to either enhancement or suppression of cortical responses.

#### 3.1.1. rTMS

rTMS protocols are generally categorized into high-frequency (>5 Hz) and low-frequency (≤1 Hz) stimulation, which increase and decrease neuronal excitability, respectively [73]. In human studies, HF-rTMS enhances the size of MEPs during and after stimulation trains, consistent with its design to promote cortical excitation and synaptic potentiation (LTP-like plasticity) [74]. Conversely, LF-rTMS reduces MEP amplitudes and is typically used to induce cortical inhibition and synaptic depression (LTD-like plasticity) in targeted brain regions [75]. Findings from animal models broadly confirm these changes in cortical excitability and have begun to elucidate the underlying cellular and molecular mechanisms.

Several studies converge on the role of BDNF and ERK signaling. Baek and colleagues demonstrated that in N2a cell cultures exposed to ischemia/reperfusion (I/R) injury, 10 Hz—but not 0.5 Hz—stimulation activated ERK, while both frequencies increased BDNF expression [42]. Similarly, in a rat depression model, three weeks of 15 Hz rTMS enhanced BDNF and ERK levels, with effects persisting two weeks beyond stimulation [43]. In AD models, McNerney and coworkers reported that 10 Hz rTMS delivered for two to six weeks upregulated BDNF expression and cholinergic signaling [48], while Kim and colleagues showed that both 1 Hz and 10 Hz protocols promoted proliferation (BDNF), differentiation (CREB), survival (ERK, STAT3, STAT5), and anti-apoptotic signaling (Bcl-2, Akt), albeit with longer-lasting effects at 10 Hz [47].

Other work has described the synaptic and apoptotic pathways. In Sprague Dawley rats, repeated 1 Hz rTMS over five days enhanced intracellular transport and synaptic plasticity compared to a single session, which still induced early gene and miRNA changes related to synaptic function [46]. In models of depression, 15 Hz rTMS for seven days ameliorated depressive-like behaviors and upregulated hippocampal CB1R, BDNF, and Bcl-2/Bax expression [51]. Stroke models have provided converging evidence: Gao and colleagues showed that seven days of 20 Hz rTMS increased the Bcl-2/Bax ratio in a rat middle cerebral artery occlusion (MCAO) model [44], while Guo and colleagues reported enhanced BDNF-TrkB signaling and a higher Bcl-2/Bax ratio following 14 days of 10 Hz stimulation [45].

Epigenetic modulation has also emerged as a possible mechanism. Meneses-San Juan and coworkers found that four weeks of 5 Hz rTMS in a female mouse depression model increased synaptophysin (SYP), histone H3 trimethylation, and reversed stress-induced global DNA hypomethylation [49]. Complementary evidence of neurotransmission modulation comes from Weiler and colleagues, who showed that both 1 Hz rTMS and iTBS altered the expression of genes involved in GABAergic, glutamatergic, cholinergic, and calcium signaling, as well as receptor subunits, ion channels, and mitochondrial proteins in rats and hippocampal brain slices [52].

Finally, translational studies provide further support for the involvement of BDNF-related pathways. Wang and colleagues demonstrated that rTMS enhanced BDNF expression through ERK2 signaling in a rat depression model and found parallel increases in BDNF/NMDAR expression in both rat brain slices and human lymphocytes [50], suggesting conserved mechanisms across species.

#### 3.1.2. Theta Burst Stimulation

In human studies, TBS delivers short high-frequency bursts at a fixed repetition rate to induce lasting changes in cortical or cerebellar excitability [74,76,77]. Theta frequency was initially selected under the assumption that stimulation at the intrinsic rhythm of the hippocampus would promote synaptic plasticity in pyramidal neurons [74]. Depending on the delivery pattern, TBS can produce excitatory or inhibitory effects: intermittent TBS (iTBS) facilitates LTP-like plasticity, whereas continuous TBS (cTBS) induces LTD-like inhibition. iTBS consists of 50 Hz bursts delivered at 5 Hz intervals in 2 s trains every 10 s for 190 s, while cTBS applies the same bursts continuously for 40 s. Both protocols consist of 600 pulses and can elicit effects lasting over an hour, but within a much shorter stimulation time compared to conventional rTMS [74,78,79].

Animal studies have identified calcium signaling as a central mechanism of iTBS-mediated plasticity. Gandolfi and colleagues applied TBS (8 bursts of 10 pulses at 100 Hz every 250 ms) to rat cerebellar slices and observed increased expression of CREB and c-Fos, downstream effectors of NMDAR activation; these effects were abolished by NMDAR antagonists, confirming their involvement [54]. Similarly, Labedi and coworkers demonstrated that iTBS-induced suppression of inhibitory interneurons is mediated by NMDA receptors: ketamine at sub-narcotic doses partially attenuated, and at higher doses completely abolished, the reduction in GAD67 and parvalbumin (PV) expression, while suppression of calbindin (CB) expression persisted, suggesting distinct mechanisms for PV+ versus CB+ interneurons [57].

Additional in vitro work supports broader molecular effects. Ismail et al. applied iTBS to N2a neuroblastoma cells and found upregulation of calcium-, GABA-, and glutamate-related subunits, along with increased BDNF and TrkB expression [56]. Consistently, Stekic and coworkers showed that iTBS enhanced phosphorylation of ERK1/2 and PI3K and restored mTOR and Akt levels in an AD rat model, indicating modulation of the BDNF pathway [59]. In MCAO-induced rats, Ljubisavljevic and colleagues compared iTBS, cTBS, and rTMS (1 Hz and 5 Hz) and found that while BDNF expression increased across several groups, only iTBS upregulated activity-dependent transcriptional regulators of long-term plasticity, glutamatergic and GABA receptor subunits, and glutamate synthesis [58].

iTBS has also been linked to modulation of inflammation and neuroprotection. In a MCAO rat model, Hu et al. showed that 28 days of iTBS reduced glial activation, suppressed proinflammatory molecules, and enhanced anti-inflammatory signaling [55]. Finally, Wu and colleagues reported that 14 days of iTBS over the sagittal suture increased c-Fos-positive neuron density in the hippocampal CA1 region of male SD rats, further supporting the role of iTBS in activating plasticity-related pathways [61].

#### 3.1.3. PAS

PAS combines activation of a sensory pathway, typically via electrical stimulation of a peripheral nerve, with TMS applied over the primary motor cortex (M1) to induce spike timing-dependent plasticity (STDP) [80,81,82]. When the afferent input from peripheral stimulation reaches the cortex just before the TMS pulse, PAS can enhance cortical excitability in a temporally specific and long-lasting manner, mimicking LTP-like plasticity. In human studies, PAS effects are abolished by NMDA receptor antagonists such as dextromethorphan, memantine, and D-cycloserine, underscoring the critical role of NMDARs in PAS-induced plasticity [83,84,85]. Conversely, acetylcholinesterase inhibitors such as rivastigmine enhance both LTP- and LTD-like effects of PAS [86].

A notable translational study by Battaglia and colleagues demonstrated altered NMDA-dependent plasticity across M1, the prefrontal cortex, and the hippocampus of APP/PS1 transgenic mice after TBS/HF-rTMS. These findings were paralleled by impaired PAS plasticity in patients with moderate AD, highlighting the conserved mechanisms of NMDAR-dependent modulation across species [53].

### 3.2. tES

#### 3.2.1. tDCS

tDCS delivers constant current across the scalp between an anode and a cathode, with the goal of subthreshold modulation of the resting membrane potential of cortical neurons. This produces after-effects on cortical excitability that can persist from minutes to hours [87]. The direction of current flow is critical: anodal stimulation generally increases excitability, whereas cathodal stimulation reduces it. However, responses also depend on the morphology of the stimulated area [77,88,89,90].

Glutamatergic signaling is a key mechanism mediating tDCS effects. NMDA receptor activation is required for sustaining tDCS-induced plasticity, and calcium-dependent pathways mediate its non-linear dose–response [62,91,92,93,94]. Monoaminergic systems also modulate these effects through dopaminergic, serotonergic, and noradrenergic signaling, although human pharmacological studies mainly confirm these pathways’ modulatory influence [95,96].

Evidence from animal and cellular models highlights BDNF as another key mediator. BDNF secretion depends on calcium and NMDA receptor activation [97,98,99] and supports neuronal regeneration and LTP. In a mouse stroke model, tDCS increased peri-infarct BDNF levels and spike firing, with corresponding increases in circulating BDNF that predicted functional recovery [66]. In vitro, DCS-induced synaptic potentiation was absent in BDNF-deficient brain slices [100], and stimulation of astrocytes alone increased BDNF gene expression [63]. These findings align with computational and experimental evidence implicating glial polarization in tDCS effects [71,101]. Beyond plasticity, tDCS has been shown to modulate autophagy and reduce α-synuclein oligomers in neuroblastoma cell lines [102]. Translationally, BDNF genotype has been linked to tDCS efficacy in stroke-related aphasia recovery [103], though not in depression [104].

tDCS may also support neuroregeneration. In mouse models of cardiac arrest-induced neuronal death, tDCS enhanced expression of proteins related to synaptic function and regeneration, including MAP2, GAP43, PSD95, and synaptophysin [71,105]. Evidence of trans-synaptic neurogenesis in the dorsal hippocampus following frontal cortex stimulation suggests effects extending to interconnected regions [106].

Finally, peripheral contributions cannot be excluded. tDCS modulates tES-evoked potentials, thought to be independent of cortical synaptic activity, as well as compound muscle action potentials from peripheral nerve stimulation, in humans [107]. These effects may reflect arousal-related activation of the ascending reticular activating system. A translational study by Sun and colleagues identified a novel cathodal tDCS-related pathway in mouse brain slices that persisted despite GABAergic and glutamatergic blockade, and found comparable effects in resected human epileptogenic tissue [70].

#### 3.2.2. tACS

Unlike tDCS, tACS does not directly induce synaptic plasticity. Instead, it delivers a weak alternating current (1–2 mA) that modulates brain activity through the “resonance principle,” entraining cortical oscillations and influencing neuronal firing patterns without changing the overall firing rate. By using a sinusoidal waveform, tACS can synchronize or desynchronize neuronal networks, with effects determined by stimulation parameters such as frequency, intensity, and phase alignment [87,89,108].

Although not directly plasticity-inducing, alternating electric fields can transiently modify synaptic activity by altering presynaptic calcium dynamics, thereby providing a substrate for downstream neuromodulatory effects. Consistent with this, several studies have shown that tACS interacts with multiple neurotransmitter systems, suggesting potential therapeutic applications in rebalancing excitatory and inhibitory signaling [14,109]. For example, the after-effects of tACS have been linked to NMDA receptor-dependent plasticity [110]. Moreover, it has been shown to restore cholinergic–adrenergic balance after pharmacological perturbation with reserpine (an anti-adrenergic drug) and physostigmine (a reversible cholinesterase inhibitor) [111]. In addition, tACS can influence serotonergic transmission, both by enhancing 5-HT synthesis in the hippocampus, frontal cortex, and hypothalamus [112] and by modulating activity within brainstem nuclei such as the raphe, locus coeruleus, and laterodorsal/pedunculopontine cholinergic nuclei [109].

## 4. Discussion

**Figure 2:**
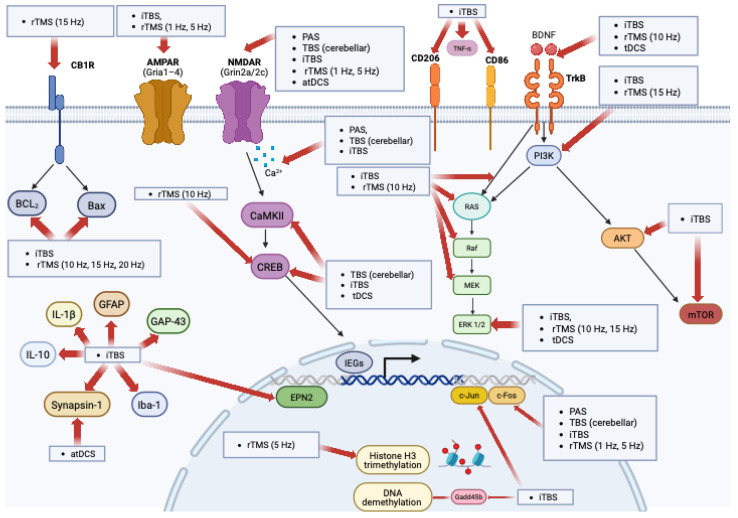
Cellular and molecular brain plasticity pathways and associated brain stimulation techniques.

This systematic review provides a comprehensive and up-to-date overview of the molecular and cellular underpinnings of the most commonly applied NIBS techniques (Figure 2), in accordance with the PRISMA 2020 guidelines (Supplementary Table S1) [113]. Evidence was gathered from in vivo, in vitro, and ex vivo studies using healthy animals, disease models, and cellular preparations. While many investigations have examined the cellular and molecular effects of TMS and tES, findings are not entirely consistent, and successful translation from animal models to humans remains limited. These inconsistencies likely reflect methodological variability in stimulation parameters, experimental models, and outcome measures, which should be considered when interpreting the overall evidence.

To date, only preliminary and partial cross-species evidence supports the existence of shared molecular mechanisms of NIBS. Wang and colleagues [50] showed that high-frequency rTMS modulates the BDNF–TrkB–NMDAR pathway—including ERK2, PI3K, and Akt activation—in both rat tissue and human lymphocytes; however, these results do not establish downstream engagement of mTOR in humans. Battaglia and coworkers [53] reported altered NMDA-dependent plasticity in APP/PS1 transgenic mice and in patients with AD, with convergent changes in NR2A/NR2B phosphorylation and PSD-95 expression, using PAS and TBS. Sun and coworkers [70] identified mGluR5–mTOR signaling as a novel pathway underlying cathodal DCS-induced LTD, validated in mouse brain slices and resected human cortical tissue, though this work did not show BDNF-dependent initiation of the pathway in human samples. Together, these findings support the possibility that NIBS can modulate partially conserved molecular pathways across species, while also underscoring important gaps: no study has yet validated the full BDNF→mTOR cascade in humans, and systematic translational research is still needed to determine how closely NIBS-induced plasticity mechanisms align between animal models and the human brain.

Across studies, molecular and biochemical modulation induced by NIBS appears to converge on distinct but interconnected pathways. Among these, BDNF emerges as a central hub. Its regulation has been documented with iTBS [56,58], cTBS [58], rTMS at different frequencies (5–15 Hz) [43,45,48,50,51,58], and tDCS [66,71]. In particular, several studies [47,63,66,68] demonstrated that anodal tDCS (atDCS) enhances BDNF expression with downstream activation of CREB and CaMKII. These excitatory protocols rely on calcium influx—mainly through NMDA receptors and voltage-gated calcium channels—initiating signaling cascades that promote BDNF transcription and synaptic remodeling. Supporting this view, Longo and coworkers [66] showed that atDCS in a stroke model increased CaMKII and CREB levels, pointing to a calcium-dependent pathway mediating neuroplasticity.

Further downstream, Stekic and colleagues [59] and Wang and coworkers [50] reported that 15 Hz rTMS and iTBS enhance phosphorylation of PI3K and ERK1/2 and increase Akt and mTOR levels, all downstream effectors of BDNF–TrkB signaling. While these results support BDNF as an endogenous ligand initiating synaptic plasticity, Longo and colleagues [66] suggested that atDCS may instead preferentially engage a BDNF → TrkB → ERK1/2 route. This divergence indicates that although NIBS techniques may converge on BDNF–TrkB signaling, they diverge in their downstream cascades, potentially leading to distinct forms of structural and functional plasticity.

Glutamatergic transmission is another key target of NIBS. Glutamate acts through ionotropic receptors (NMDA, AMPA, kainate) involved in fast excitatory transmission and early synaptic modifications, and through metabotropic receptors (mGluRs) that support long-term plasticity and neuromodulation [56,58,61,114]. Wu and coworkers [61] showed that iTBS increases glutamate levels and reduces the GABA/Glu ratio, suggesting a shift toward excitation. This was accompanied by upregulation of AMPA receptor subunits (Gria1–4) and NMDA receptor subunits (Grin2a–c) [58], pointing to reinforcement of both fast synaptic transmission and calcium-dependent plasticity. Importantly, AMPA receptor upregulation facilitates rapid excitatory signaling, whereas NMDA receptor modulation supports longer-term synaptic remodeling [42,58,61,115].

Interestingly, BDNF and NMDA signaling appear to converge under certain conditions. Studies on atDCS [47,66] demonstrated increased BDNF expression and ERK1/2 activation, a cascade often linked to NMDA receptor function. Given that NMDA receptors themselves can trigger ERK1/2 activation [116], these findings suggest that BDNF and NMDA pathways may converge on common intracellular mechanisms of plasticity, amplifying each other’s effects.

Inhibitory neurotransmission is also shaped by NIBS. GABAergic modulation appears selective and context-dependent: iTBS reduces GABA levels [61], ctDCS increases GAD65/67 with an interhemispheric imbalance [117], and iTBS decreases GAD67 and parvalbumin but not calbindin, with differential NMDA dependence across interneuron subtypes [57]. These findings indicate that NIBS can differentially modulate inhibitory circuits, with implications for cortical reorganization.

At the transcriptional level, NIBS activates multiple genes involved in synaptic plasticity, including c-Fos, Arc [68], CREB [47,68], CaMKII [47,66], PSD-95 [66], Synapsin I [47], and others regulating structural remodeling and neuroprotection [60]. While these results collectively show the capacity of NIBS to engage broad genetic programs, their efficacy may be compromised in pathological contexts. For instance, Battaglia and colleagues [53] reported altered NR2A/NR2B subunit composition and absent PAS-induced potentiation in Alzheimer’s patients, highlighting impaired NMDA receptor function as a bottleneck for plasticity.

Taken together, the evidence suggests that NIBS influences a rich and interconnected network of molecular targets—including BDNF, glutamate, GABA, and plasticity-related genes. Although different techniques share some common molecular triggers, they diverge in downstream signaling, leading to distinct functional outcomes. The challenge ahead lies in clarifying these convergences and divergences, and in validating molecular mechanisms across species and clinical populations, in order to fully harness the therapeutic potential of NIBS.

Although several of the pathways discussed—particularly those involving BDNF–TrkB signaling—are hypothesized to contribute to recovery in neurodegenerative and cerebrovascular disorders, current evidence remains largely preclinical, and consistent links between molecular modulation and clinical symptom improvement have yet to be demonstrated. Accordingly, drawing therapeutic conclusions would be premature.

### Future Directions and Limitation of Translation in Humans

The translation of NIBS research from preclinical models to human applications remains challenging. A key barrier is that, although NIBS is often interpreted within a simplified LTP/LTD framework, current evidence shows that it engages broader and more dynamic forms of plasticity, including modulation of glutamatergic signaling, BDNF–TrkB pathways, intracellular cascades (ERK, PI3K/Akt, mTOR), epigenetic regulation, neuroinflammatory processes, interneuron-specific GABAergic signaling, neurogenesis, and structural synaptic remodeling. Because these effects extend beyond binary potentiation or depression, future research must employ approaches capable of capturing multi-level plasticity changes rather than focusing solely on excitability shifts. Translational progress is further limited by incomplete mechanistic understanding and inconsistent outcome measures across animal and human studies. The wide range of stimulation parameters available for both tDCS and rTMS further complicates interpretation. Given the emerging evidence for shared molecular and cellular targets across NIBS modalities, preclinical research should prioritize evidence-based frameworks for cortical target selection and the establishment of direct causal links between stimulation, target engagement, and biomarker modulation [118].

Future clinical studies would benefit from integrating multimodal biomarkers, including MEPs, TMS-EEG, EEG, and fMRI, allowing quantification of plasticity-related changes across circuit levels and enabling comparison across protocols [118,119]. Such approaches are necessary because relying only on excitability biomarkers risks overlooking broader adaptations involving neuroimmune, epigenetic, and metabolic pathways.

A persistent obstacle is the complexity of NIBS-induced neural activation: stimulation recruits large neuronal populations, including both inhibitory and excitatory neurons, making it difficult to extrapolate from in vitro models designed for highly controlled synaptic plasticity paradigms [118]. Computational modeling has improved estimation of electric field distributions and interactions with neuronal compartments, [120], yet uncertainty remains regarding whether axons, somas, dendrites, or glial-associated structures are most affected [120,121]. Recognition that NIBS likely influences neuron–glia interactions and neuroinflammatory signaling supports the need for experimental systems that reflect cellular diversity, including co-cultures of neurons, astrocytes, and microglia, which are better suited for studying glial contributions to synaptic and structural plasticity [89,122].

Beyond synaptic efficacy, structural plasticity, including changes in dendritic spine density, synapse formation, dendritic remodeling, and axonal sprouting, is a promising target given its relevance to long-term reorganization of neural networks and recovery of function [15]. Advances in in vivo microscopy and live-cell imaging now permit real-time visualization of plasticity events at subcellular resolution, offering an opportunity to directly link molecular signaling with circuit remodeling over time. Future research should prioritize experimental systems that capture this cellular diversity.

Finally, meaningful translation requires continued parallel progress in preclinical and clinical research. Although convergent molecular pathways across rodents, humans, and induced pluripotent stem cell-derived neural cultures support translational potential, species-specific differences in cortical architecture necessitate iterative cross-validation of mechanisms [118,122]. Integrating mechanistic insights with human neurophysiological and imaging data will be essential to closing the translational gap and defining the therapeutic potential of NIBS across neurological and psychiatric diseases.

## 5. Conclusions

In this systematic review, we examined how NIBS induces molecular and cellular changes linked to neural plasticity and considered whether these effects may arise from shared or modality-specific pathways. Across studies, several recurring patterns emerged, including consistent influences on key neurotransmitter systems and intracellular signaling cascades. Although the evidence base is heterogeneous and some modalities remain better studied than others, the overall findings indicate that different forms of NIBS tend to engage overlapping molecular processes. Beyond the traditional view of NIBS protocols as producing LTP-like or LTD-like effects, the molecular data point to broader adaptive responses involving epigenetic regulation, modulation of neuroinflammatory pathways, interneuron-specific GABAergic changes, neurogenesis, and synaptic remodeling. These observations suggest that NIBS does not act through a single, binary synaptic mechanism but instead influences multiple levels of plasticity. This molecular perspective complements neurophysiological measures such as MEPs and helps to bridge the gap between observed functional effects and their underlying biological substrates. By integrating findings on neurotransmitter dynamics, gene expression, and intracellular signaling, this review supports a system-level view of how NIBS shapes neuroplasticity while recognizing that further standardized and translational studies—particularly in humans—will be important to refine and extend this emerging mechanistic framework.

## Figures and Tables

**Table 1:** Search strategy. The left column lists NIBS techniques, which are combined using Boolean operators with the terms in the central (mechanisms) and right (models/populations) columns.

“repetitive transcranial magnetic stimulation”[Title/Abstract] OR “rTMS”[Title/Abstract] “theta burst stimulation”[Title/Abstract] OR “TBS”[Title/Abstract] ------------------------------------------- “paired associative stimulation”[Title/Abstract] OR “PAS”[Title/Abstract] ------------------------------------------- “transcranial direct current stimulation”[Title/Abstract] OR “tDCS”[Title/Abstract] ------------------------------------------- “transcranial alternating current stimulation”[Title/Abstract] OR “tACS”[Title/Abstract]	AND	“cellular effects”[Title/Abstract] OR “molecular effects”[Title/Abstract] OR “mechanisms”[Title/Abstract] OR “cellular mechanisms”[Title/Abstract] OR “molecular mechanisms”[Title/Abstract] OR “neuroplasticity”[Title/Abstract] OR “gene expression”[Title/Abstract] OR “synaptic plasticity”[Title/Abstract] OR “LTP-like”[Title/Abstract] OR “LTD-like”[Title/Abstract])	AND	“human”[Title/Abstract] OR “ex vivo”[Title/Abstract] OR “in vivo”[Title/Abstract] OR “animal model”[Title/Abstract] OR “murine”[Title/Abstract] OR “primate”[Title/Abstract] OR “mammalian”[Title/Abstract] OR “cell line”[Title/Abstract] OR “neuron”[Title/Abstract] OR “in vitro”[Title/Abstract] OR “neurodegenerative disease”[Title/Abstract] OR “Alzheimer’s Disease”[Title/Abstract] OR “Parkinson’s Disease”[Title/Abstract] OR “stroke”[Title/Abstract] OR “psychiatric diseases”[Title/Abstract]

**Table 2:** Characteristics of retrieved rTMS studies (listed in alphabetical order). rMS, repeated magnetic stimulation; I/R, ischemic/riperfusion; OGD/R, Oxygen glucose deprivation/reoxygenation; qRT-PCR, quantitative real-time polymerase chain reaction; ICC; immunocytochemistry; CaMKII-CREB, Ca^2+^-calmodulin-dependent protein-kinase II-cAMP-response element binding protein; ERK, extracellular regulated kinases; BDNF, brain-derived neurotrophic factor; SYN-1, synaptophysin 1; rTMS, repeated transcranial magnetic stimulation; CUMS, chronic unpredicted mild stress; SD, Sprague-Dawley; Ven, venlafaxine; MCAO, middle cerebral artery occlusion; IHC, immunohistochemistry; IF, immunofluorescence; STZ, streptozocin; AD, Alzheimer disease; ELISA, enzyme-linked immunosorbent assay; FC, frontal cortex; DG, dentate gyrus; MSO, maximum stimulator output; RMT, resting motor threshold; HC, healthy controls; CSF, cerebrospinal fluid; CB1R, cannabinoid type 1 receptor; iTBS, intermittent theta-burst stimulation; FLX, fluoxetine; PFC, prefrontal cortex; CB1R, cannabinoid-1 receptor; KAR, kainate receptors.

Authors	Stimulation Technique and Protocol	Parameters/Site of Stimulation	Animal Model/Neural Substrate	N° Groups/Participants	Cellular/Molecular/Genetic Techniques USED	Translation to Human Studies	Results
Baek et al., 2018 [42]	rMS	0.5/10 Hz, 10 min of stimulation	Mouse N2a cell culture of I/R model injury (OGD/R)	3 groups: OGD/R + sham, OGD/R+LF (0.5 Hz) and OGD/R+HF (10 Hz)	qRT-PCR, Western blot, ICC	No	OGD/R+LF: ↓ p-ERK and p-AKT, ↓ BAX and caspase-3, ↑ Bcl-2 and Pro-caspase-3, ↓ NMDAR1, CaMKII–CREB OGD/R+HF: ↑ p-ERK and p-AKT, ↑ BAX and caspase-3, ↓ Bcl-2 and Pro-caspase-3, ↑ NMDAR1, CaMKII–CREB, ↑ BDNF, SYN-1 and PSD-95
Feng et al., 2012 [43]	rTMS	15 Hz, 1000 pulses/d, 3 w, 100% MSO, vertex	Depression model (CUMS) in male SD rats	84 divided in 7 groups: sham, rTMS, Ven, CUMS, CUMS + rTMS, CUMS+Ven, CUMS + rTMS + Ven	ICC, Western blot, ELISA	No	↑ BDNF and pERK1/2 after 3 weeks of rTMS and continued to stay at a stable high level 2 weeks later, after the treatments stopped
Gao et al., 2010 [44]	rTMS	20 Hz, 5 s × 10 times, 7 d, right fronto-parietal cortex (bregma)	SD rat models of MCAO-stroke	30, divided into 3 groups: control, rTMS, sham.	IHC	No	↓ caspase3, ↑ Bcl-2 and ↑ Bcl-2/Bax ratio in the rTMS group
Guo et al., 2017 [45]	rTMS	10 Hz, 10 times (300 pulses/d), 120% RMT, bregma	male SD rat models of MCAO	7- and 14-day-treatment groups, divided into sham, MCAO and rTMS groups.	IF, Western blot, qRT-PCR,	No	↑ BDNF, TrkB, p-AKT and Bcl-2 protein expression, and ↓ Bax expression in hippocampus during rTMS
Hwang et al., 2022 [46]	rTMS	1 Hz single vs. repeated session (20 min/5 d), left hemisphere, 50% MT	male SD rats	16 rats. Single session: 4 real stim, 4 sham Repeated session (5 d): 4 real stim, 4 sham	mRNA-miRNA microarray analysis	No	+ regulation of intracellular transport and synaptic plasticity only with repeated rTMS group. A single session of rTMS primarily induced changes in the early genes.
Kim et al., 2024 [47]	rTMS	1/10 Hz. - Cell lines: 6 cycles,30 min, 3 times, 2 d - Animal: 20 min,1 d, 4 w	STZ-induced model of AD in human neuroblastoma cell line. Male SD rats’ hippocampi	15 rats divided into 3 groups: control, sham rTMS on STZ-induced AD and real rTMS on STZ-induced AD	IHC, qRT-PCR, Western blotting	No	↑ STAT1, STAT3, STAT5, ERK, JNK, Akt, p70S6K, and CREB in cell lines and in AD’s animal model after 10 Hz rTMS ↑ ERK, JNK, Akt, p70S6K in 1 Hz and in 10 Hz rTMS groups at 20 min after stimulation. ↑ CREB only in 10 Hz rTMS after 20 min. Phosphorylation lasts 3 h in 10 Hz and 1 h in 1 Hz rTMS.
McNerney et al., 2022 [48]	rTMS	10 Hz, 10 min/d, 2 times/w, 6 w, bregma	Female 3xTgAD mice and their wild type controls	103 mice divided into real and sham, 2 weeks- and 6 weeks-stimulation groups	IHC, qRT-PCR, ELISA	No	= BDNF in the wild-type group that received 2 weeks of rTMS and ↑ in the 6-week group. ↑ BDNF expression in the 2-week and 6-week rTMS in 3xTgAD groups
Meneses-San Juan et al., 2023 [49]	rTMS	5 Hz, 5 d × 4 w, 1500 pulses/d, FC and DG stimulation	Female BALB/c mice model of depression (CUMS)	40 mice, divided in 2 groups: real rTMS and control group (CUMS+FLX)	IHC, IF, ELISA	No	5 Hz rTMS and FLX reverse the decreased density of the DSs in the FC and DG caused by the CUMS protocol. ↑ SYP in the FC of mice treated with 5 Hz rTMS or FLX. ↑ Histone acetylation and demethylation
Wang et al., 2011 [50]	rTMS	5 Hz, 5 d, 1600 pulses, 50% MSO in rats, 90% RMT in HC in M1, 1200 pulses	Ex vivo male SD rats, HC	12 rats and 8 HC, divided into 2 groups: real vs. sham.	IHC, Western blot	Yes	↑ BDNF, PLC-γ1, shc/N-shc, NMDAR subunits, PSD-95, ERK2, PI3K, Akt in brain slices of rat’s PFC and in lymphocytes
Wang et al., 2014 [51]	rTMS	15 Hz, 15 trains of 60 pulses, 100% MSO, 7 d, vertex	Depression model (CUMS) in male SD rats	36 rats, divided in 4 groups: sham, sham+rTMS, CUMS, CUMS+rTMS	Western blotting, ICC	No	↑ CB1R, BDNF and Bcl-2/Bax expression levels in the hippocampus after rTMS ↑ CB1R, abolished after administration of a CB1R antagonist
Weiler et al., 2023 [52]	rTMS (1 Hz), iTBS	15% MSO. rTMs and iTBS: 5 blocks, 600 pulses, repeated at 15 min intervals.	Ex vivo and in vivo male Long–Evans rats. In vitro hippocampal neurons of SD rats	12 Long–Evans rats and 16 SD rats	microarray-based gene expression	No	- In the ex vivo and in vivo Long–Evans model: ↑ Ptk2b, Slc6a13, ↑ Slc5a7, ↑ Ryr2, Chrna5, Grin3a, Glun3a, Arc, Cnp. - In vitro SD rat model: ↑ Gabbr1,2 and Gabra4; ↑ Grik1,4; ↑ Grm3–7.

**Table 3:** Characteristics of retrieved TBS studies (listed in alphabetical order). TBS, theta-burst stimulation; IHC, immunohistochemistry; IF, immunofluorescence; CREB, cAMP response element-binding protein; iTBS, intermittent theta-burst stimulation; I/R ischemia/reperfusion; MCAO, middle cerebral artery occlusion; SD, Sprague-Dawley; qRT-PCR, quantitative real-time reverse transcription polymerase chain reaction; ELISA, enzyme-linked immunosorbent assay; GFAP, glial fibrillary acidic protein; MSO, maximum stimulator output; cTBS, continuous theta-burst stimulation; AD, Alzheimer Disease; TMT, trimethyltin; ISI, inter-stimulus interval; RMT, resting motor threshold; PT, phototrombotic; PAS, paired-associative stimulation; APP, amyloid precursor protein; PS1, presenilin-1; M1, primary motor cortex; PFC, prefrontal cortex, DG, dentate gyrus; WT, wild-type.

Authors	Stimulation Technique and Protocol	Parameters/Site of Stimulation	Animal Model/Neural Substrate	N° Groups/Participants	Cellular/Molecular/Genetic Techniques Used	Translation to Human Studies	Results
Battaglia et al., 2007 [53]	TBS, HF-rTMS PAS	AD patients: - PAS protocol (200 stimuli, ISI 25 msec); transgenic mice: - TBS (50% MSO) in M1, - HF-rTMS in PFC (300 Hz) and in DG (100 Hz)	AD patients and double transgenic mice (APP/PS1)	10 AD patients; transgenic mice compared with WT	IHC, Western blot	Yes	No increase of MEP amplitude of AD patients after PAS ↓ tyrosine-phosphorylated NR2A/NR2B in APP/PS1 transgenic mice ↑ NR2A subunit only in APP/PS1 prefrontal cortex = PSD-95 in the three cortical areas of APP/PS1 mice
Gandolfi et al., 2017 [54]	TBS	8 bursts of 10 pulses at 100 Hz repeated every 250 ms	Cerebellar slices of Wistar rats	4 groups: controls, 15 min and 120 min from stim, NMDAR antagonist	In situ hybridization, IHC, IF	No	↑ P-CREB at 15 min and 120 min after TBS. ↑ c-Fos only at 120 min after TBS. No differences in P-CREB/c-Fos in the presence of an NMDAR antagonist
Hu et al., 2023 [55]	iTBS	10 bursts, 600 pulses, 28 d, 26% MSO	Cerebral I/R injured model (MCAO) in SD rats	4 groups: sham (n = 16), I/R 24 h (n = 19), I/R 28 d (n = 19), I/R + iTBS 28 d (n = 19)	IF, qRT-PCR, ELISA, Western blot, RNA transcriptome sequence analysis	No	↑ GAP-43, MMP9. ↓ GFAP, Iba-1 ↓ CD86, IL-1b, TNF-a; ↑ CD206, IL-10. ↓ CytC, caspase-3, ↑ Bcl-2. ↓ Bax, caspase-3, CytC, caspase-9 after 28 days of iTBS.
Ismail et al., 2024 [56]	iTBS	300 pulses, 25, 50, 75 and 100% MSO	N2A mouse neuroblastoma cells	12-well plate of 10^4^ cells divided into 5 groups: after 0.5 h, 3 h, 6 h, 12 h and 24 h	Immunoblotting, ICC	No	↑ NMDAR1, GABBR2, mGluR, TrkB, GAP-43, synapsin-1, BDNF and β-tubulin III at 0.5 h post-iTBS
Labedi et al., 2014 [57]	iTBS	5 blocks, 600 pulses/15 min, 23–25% MSO	male SD rats	16 rats, divided in 4 groups: sham-iTBS, real iTBS, iTBS low dose of ketamine or iTBS high dose of ketamine.	IHC	No	↓GAD67, CB and PV ketamine largely prevented the loss of PV and GAD67 expression at both low and high dose
Ljubisavljevic et al., 2015 [58]	iTBS, cTBS rTMS (1/5 Hz)	30% MSO, 1 t/d, 4 blocks, 2400 pulse/d	stroke model (MCAO) of male Wistar rats	149 rats divided in: 1 Hz, 5 Hz, iTBS, cTBS, sham	IHC, qRT-PCR	No	↑ BDNF in 5 Hz rTMS, cTBS and iTBS. ↑ Creb1, Gria1–4, Grin2a–2c, Gabbr1, ↑Gadd45b, Junb, Gls, Bai1. c-Fos and Jun ↑ only after iTBS. ↑ Plat (tPA gene) after cTBS
Stekic et al., 2022 [59]	iTBS	33% MSO, 15 sessions/2 stim per d/600 pulses, frontal	male Wistar rats of AD model induced by TMT intoxication	54 rats divided into 4 groups: - control (n = 12), - TMT (n = 15), - TMT+iTBS (n = 12) - TMT+sham (n = 15)	IHC, Western blot	No	↑ P-ERK 1/2 and PI3K in iTBS group. Restored levels of mTOR and p-Akt/compared to TMT group
Thomson et al., 2020 [60]	iTBS, cTBS	600 pulses, 100% MSO	in vitro SH-SY5Y human neuron model	3 conditions: - cTBS, - iTBS, - sham collected after stim or at 6 h or 24 h	qRT-PCR, IF	No	↑ NTRK2, Bcl2 and MAPK9 after 24 h of iTBS. No effects of cTBS on gene expression
Wu et al., 2024 [61]	iTBS	20 trains, 600 pulses, 14 d, 30% MSO or 80% RMT	male SD rats	97 rats divided into 2 groups: real and sham	IHC, microdyalisis	No	↑ c-Fos. Normalized theta power significantly higher in real iTBS group. ↓ GABA, ↑ Glu, ↓ GABA/Glu ratio in real iTBs group

**Table 4:** Characteristics of retrieved tDCS studies (listed in alphabetical order). atDCS, anodal transcranial direct current stimulation; SD, Sprague-Dawley; G6P, glucose-6-phosphate; 3-BAIBA, D,L-3-aminoisobutyric acid; TCA cycle, tricarboxylic acid cycle; DCS, direct current stimulation; HA, human astrocytes; EC, endothelial cells; qRT-PCR, quantitative real-time reverse transcription polymerase chain reaction; NOS3, nitric oxide synthase 3; BBB, blood–brain barrier; BDNF, brain derived neurotropic factor; NGS, next-generation sequencing; CREB, cAMP-response element binding protein; CaMKII, Ca^2+^-calmodulin-dependent protein-kinase II; PT, phototrombotic; M1, primary motor cortex area; WT, wild-type; miRNA, microRNA; lncRNA, long non-coding RNA; circRNA, circular RNA; tACS, transcranial alternating current stimulation; SEP, somatosensory-evoked potential; ctDCS, cathodal transcranial direct current stimulation.

Authors	Stimulation Technique and Protocol	Parameters/Site of Stimulation	Animal Model/Neural Substrate	N° Groups/Participants	Cellular/Molecular/Genetic Techniques Used	Translation to Human Studies	Results
Agrawal et al., 2024 [62]	atDCS	Parietal cortex, 20 min, 5 d, 250 μA	Male SD rats	6 rats	mRNA sequencing and Metabolomic Analysis	No	↑ adenosine, G6P, 3-BAIBA, ↓ sphingosine. ↑ glycolysis and mitochondrial function by the TCA cycle. ↓ Ca^2+^-related signal.
Cancel et al., 2022 [63]	DCS	0.1–1 mA for 10 min	In vitro HA and mouse brain EC	A monolayer of 3 × 10^4^ HA/cm^2^ and 6 × 10^4^ EC/cm^2^	qRT-PCR and Western blot immediately and 1 h after stim	No	↑ NOS3 and VEGFR1 (modulate permeability of BBB ↑ c-FOS and BDNF in astrocytes
Holmes et al., 2016 [64]	atDCS	sham, 250- 500- 2000 μA 20 min, sagittal suture, 2.5 mm caudal bregma	Male SD rats	7–8 rats × group stim condition	NGS whole transcriptome RNA-sequencing analysis	No	↑ signaling pathways related to Ca^2+^ ion binding, transmembrane/signal peptide and NLRP3- IL-1*β* pathway. ↑ Ras signaling pathway
Kim et al., 2017 [65]	atDCS	250 μA, 20 min, 7 d in the right sensorimotor cortex	Male SD rats	19 rats, divided into 3 groups: intact control group(n = 5), sham-operated group (n = 7), real stim group (n = 7)	qRT-PCR after 6 h of stim	No	↑ NMDAR and BDNF, CREB, CaMKII, and synapsin I. ↑ c-Fos and Arc.
Longo et al., 2022 [66]	tDCS delivered with 2 epicranial electrodes	72 h post PT stroke on M1, 3 sessions of 250 μA, 20 min,3 d	C57BL/6 male mice	Not described. Divided into 2 groups: real or sham tDCS	Western immunoblotting, qRT-PCR, ELISA 24 h after stim	No	↑ ERK1/2-CREB, CaMKII, BDNF. ↑ PSD-95
Magri et al., 2021 [67]	atDCS with unilateral epicranial electrode	HP (3 stim, 20 min, 3 consecutive days), PFC (6 stim, 15 min, 3 d × 2 w)	3xTg-AD mouse vs. age-matched WT mice	14 real vs. 9 sham AD mouse; 7 real vs. 9 sham control WT mice	mRNA sequencing and blood whole transcriptome analysis	No	tDCS is able to modulate the gene expression of peripheral tissues, such as blood, and it suggests that blood gene expression profiles could be used as biomarkers of synaptic plasticity
Podda et al., 2016 [68]	atDCS with unilateral epicranial electrode	left hippocampi,1 mm left and 1 mm posterior to bregma, 350 μA, 20 min	Male C57 BL/6 mice	18 mice (n = 9 active stim, n = 9 sham control)	qRT-PCR and ELISA, 24 h after stim Western blot 2 h after stim	No	↑ BDNF and pCREB
Sánchez-León et al., 2021 [69]	Anodal or cathodal tDCS, tACS	tDCS: Right-S1, 20 min, 200 μA for cathodal, 150 μA for anodal. tACS: 2,20,200 μA at 1 Hz	adult male C57 mice	10 mice, divided into 2 groups: real and sham stim	IHC	No	↑ GAD65–67 and GABA level imbalance after cathodal stimulation but no changes after anodal stimulation. ↑ SEP amplitude during anodal stimulation and ↓ during cathodal stimulation
Sun et al., 2016 [70]	Cathodal DCS	In vitro: 300 or 400 μA, 10 or 25 min. In vivo: 1 mA, 25 min	In vitro brain slices of male C57BL/6 mice, human cortex in vivo (surgical removal of epileptogenic zone)	Not described	Immunoblot at 0, 15, 30, 60 min in vitro after stim	Yes	mGluR5-mTOR signaling as a novel pathway that neither GABAR nor NMDAR blockade abolished DCS-LTD
Walter et al., 2022 [71]	Anodal or cathodal tDCS	PT, bregma, 2 sessions: 15 min, 250 or 500 μA, 5 d	male C57BL/6JRj mice	62 divided into 2 groups: real or sham stim	IHC	No	ctDCS: ↑ functional recovery, neurogenesis. ↓ microglial activation, and CD16/32. atDCS ↑ neurogenesis.

## Data Availability

No new data were created or analyzed in this study.

## Supplementary Materials

The following supporting information can be downloaded at: https://www.mdpi.com/article/10.3390/cells14241996/s1, The completed PRISMA 2020 checklist is provided as Supplementary Table S1.
